# Hypertension is associated with an increased risk for severe imported falciparum malaria: a tertiary care hospital based observational study from Berlin, Germany

**DOI:** 10.1186/s12936-019-3007-4

**Published:** 2019-12-06

**Authors:** Bodo Hoffmeister, Abner Daniel Aguilar Valdez

**Affiliations:** 1 Department of Respiratory Medicine, Clinic-Group Ernst von Bergmann, Potsdam and Bad Belzig, Niemegker Straße 45, 14806 Bad Belzig, Germany; 2 Department of Endocrinology, Clinic Group Ernst von Bergmann, Potsdam and Bad Belzig, Niemegker Straße 45, 14806 Bad Belzig, Germany

**Keywords:** *Plasmodium falciparum*, Risk factors, Hypertension, Cardiovascular disease, Aging, Severe malaria

## Abstract

**Background:**

Increasing numbers of aging individuals with chronic co-morbidities travel to regions where falciparum malaria is endemic. Non-communicable diseases are now leading risk factors for death in such countries. Thus, the influence of chronic diseases on the outcome of falciparum malaria is an issue of major importance. Aim of the present study was to assess whether non-communicable diseases increase the risk for severe imported falciparum malaria.

**Methods:**

A retrospective observational study of all adult cases with imported falciparum malaria hospitalized between 2001 and 2015 in the tertiary care Charité University Hospital, Berlin, was performed.

**Results:**

A total of 536 adult patients (median age 37 years; 31.3% female) were enrolled. Of these, 329 (61.4%) originated from endemic countries, 207 patients (38.6%) from non-endemic regions. Criteria for severe malaria were fulfilled in 68 (12.7%) cases. With older age, lack of previous malaria episodes, being a tourist, and delayed presentation, well-characterized risk factors were associated with severe malaria in univariate analysis. After adjustment for these potential confounders hypertension (adjusted odds ratio aOR, 3.06 95% confidence interval, CI 1.34–7.02), cardiovascular diseases (aOR, 8.20 95% CI 2.30–29.22), and dyslipidaemia (aOR, 6.08 95% CI 1.13–32.88) were individual diseases associated with severe disease in multivariable logistic regression. Hypertension proved an independent risk factor among individuals of endemic (aOR, 4.83, 95% CI 1.44–16.22) as well as of non-endemic origin (aOR, 3.60 95% CI 1.05–12.35).

**Conclusions:**

In imported falciparum malaria hypertension and its related diseases are risk factors for severe disease.

## Background

Due to increasing international travel and changing patterns of migration malaria is still the most important tropical disease imported to Europe [1]. The pathophysiology of this protozoal disease is complex and unique and still only incompletely understood [2, 3]. In falciparum malaria, the parasite population increases 6- to 20-fold per replication cycle [4], making it the most dangerous of the five species known to affect humans. Life-threatening complications can develop rapidly and in an unpredictable manner. These complications are included in the criteria for severe falciparum malaria defined by the World Health Organization (WHO) [5].

Numerous risk factors for both severe and fatal imported falciparum malaria have been identified in recent years [6]. Among these, older age seems to play a key role, since it has consistently been found to be associated with severe malaria in multiple studies [7–10]. In the UK, for instance, the adjusted odds ratio of dying of malaria was 10.68 in those > 65 years old compared with the > 18–35 year old group [10], but the exact reasons for this association remain to be elucidated. Recently, a nationwide study from Sweden identified diabetes and obesity as two age-related, non-communicable conditions being risk factors for severe imported falciparum malaria [11]. The study also gave evidence that hypertension and cardiovascular diseases may lead to more severe courses. These observations are not only of relevance for industrialized nations, where growing numbers of older individuals with co-morbidities travel to countries where malaria is endemic [12]. Hypertension and cardiovascular diseases are now the leading risk factors for death in many low and middle-income countries where malaria is endemic [13]. Hence, the question how non-communicable diseases in general, and hypertension and cardiovascular diseases in particular, affect the risk for severe malaria, is principally an issue of major importance.

The present study aimed to identify co-morbidities associated with severe falciparum malaria imported to Berlin, Germany, in a large adult cohort. The findings are discussed in the context of the current concepts of malaria pathophysiology and in regard to the resulting implications for supportive therapy.

## Methods

### Patients

Only hospitalized patients with a first episode of acute falciparum malaria were enrolled. Since the effects of co-morbidities in an adult population should be investigated, only patients over 18 years of age were included. Diagnosis relied on thin and thick blood smears. Parasitaemia was expressed as percentage of parasitized erythrocytes with 1% parasitaemia corresponding to about 50.000 parasites/µl. Patients born in countries with malaria transmission were referred to as of “endemic origin”.

### Data collection

As part of the admission routine data on sociodemographics, travel history, chemoprophylaxis, full medical history including prior malaria episodes, current medication, and physical examination were recorded for all patients. Past medical records were retrieved, where available. Prior to statistical analysis, laboratory results and outcome parameters such as severe malaria criteria or need of intensive care treatment were retrieved. All relevant parameters were then anonymized and transferred into a database (IBM SPSS version 24).

### Severe malaria

Severe malaria was defined according to the 2014 WHO definition [5] with modifications according to [14] and [11] by presence of at least one malaria-specific complication on admission (Table 1).

**Table 1:** Criteria of severe malaria according to the 2014 World Health Organization definition with minor modifications

Criterion	Specification
Impaired consciousness	Glasgow coma scale (GCS) < 11
Multiple convulsions	> 2 convulsions within 24 h
Respiratory distress or acidotic breathing	Requirement of non-invasive or endotracheal mechanical ventilation or respiratory rate ≥ 40 breaths/min on room air
Circulatory collapse or shock	Systolic blood pressure < 80 mm Hg or ≤ 80 mm Hg despite volume repletion
Acute pulmonary oedema	Confirmed radiologically
Acute respiratory distress syndrome (ARDS)	Lung injury of acute onset, within 1 week of an apparent clinical insult and with progression of respiratory symptoms; bilateral opacities on chest imaging not explained by other lung pathology (e.g. pleural effusion, pneumothorax, or nodules); respiratory failure not explained by heart failure or volume overload; decreased arterial PaO_2_/FiO_2_ ratio (≤ 300 mmHg)
Renal impairment	Plasma or serum creatinine > 3 mg/dl (> 265 µmol/l)
Metabolic acidosis	pH < 7.25 or plasma bicarbonate < 15 mmol/l or lactate > 5 mmol/l or ≥ 45 mg/dl
Jaundice	Bilirubin > 50 µmol/l or > 3 mg/dl together with circulatory instability, respiratory distress, impaired consciousness, severe coagulopathy, or acute kidney injury
Malaria-induced anaemia	Haemoglobin level < 70 g/l or haematocrit < 20% not related to other causes than malaria
Abnormal bleeding	Including recurrent or prolonged bleeding from the nose, gums, venepuncture sites, hematemesis or melaena
Macroscopic haemoglobinuria	Macroscopic haemoglobinuria related to malaria
Hypoglycaemia	Blood glucose level < 40 mg/dl (< 2.2 µmol/l)
Hyperparasitaemia	> 5% parasitized erythrocytes

### Co-morbidity

History of or active malignancy as well as chronic cardiovascular, pulmonary, gastrointestinal, rheumatologic, renal, endocrine, metabolic, chronic infectious disorders such as hepatitis B, hepatitis C or human immunodeficiency virus (HIV) infections, psychiatric, and neurologic diseases were considered relevant chronic comorbidities. In an effort to weigh the seriousness of the underlying disorders an age-adjusted Charleson co-morbidity index (CA-CCI) [15] was calculated for every patient.

### Data analysis

Endpoint of the analysis (i.e., the dependent variable) was presence of severe malaria on admission (i.e., within the first 24 h of presentation). Categorical data were compared by χ^*2*^ test, while the Mann–Whitney-U-test was used for continuous data. Odds ratios were determined by univariate logistic regression. Based on biological plausibility, age and endemic origin were included as possible confounders in all subsequent multivariable analyses. Individual diagnoses associated with severe malaria in univariate analysis (p < 0.05) were included with potential confounders in separate multivariable models with the level of significance set at < 5%. Fit of these models was assessed by − 2 log-likelihood (comparing against the constant) and Hosmer–Lemeshow goodness-of-fit tests prior to assuring that all necessary assumptions were met (namely independence of observations, linearity in the logit for the continuous independent variables, absence of multicollinearity by using a correlation matrix and lack of significant outliers by examining the Cook’s distances) [16]. All statistical analyses were performed using IBM SPSS version 24.

### Ethics statement

The study was approved by the institutional review board (Ethics Committee of the Charité university hospital, Berlin, identifier EA1/209/18).

## Results

Between January 2001 and December 2015 a total of 558 cases of imported falciparum malaria were hospitalized in the Charité University Hospital, Berlin. Seventeen patients presented more than once. The 22 subsequent episodes of these individuals were excluded from the analysis. The remaining 536 patients ultimately enrolled represented 6.8% of all cases notified in Germany during the study period. Of these, 168 (31.3%) were female and 368 (68.7%) male. Median age of the whole patient group was 37 years (range 18–78 years). A total of 329 cases (61.4%) originated from endemic countries, 207 cases (38.6%) from non-endemic regions (Table 2). The vast majority of infections (94.8%) were contracted in sub-Saharan Africa. In 16 cases (3.0%) the disease was acquired in Asian countries. Most patients (69.6%) had no history of previous malaria episodes and had not taken regular chemoprophylaxis (79.5%). Visiting friends and relatives (VFR) was the leading reason for travel (25.1%). Sixty-eight (12.7%) cases fulfilled the criteria for severe malaria, 51 of them requiring intensive care. All 536 patients enrolled survived their infection.

**Table 2:** Sociodemographic and clinical characteristics of the study population according to disease severity

Characteristic	No. (%) of patients			P value^a^	OR (95% CI)
	Total (n = 536)	Non severe malaria (n = 468)	Severe malaria (n = 68)		Unadjusted^b^
Age, y					
Median (range)	37 (18–78)	37 (18–78)	39 (19–71)	0.07	1.023 (1.002–1.045)
Gender					
Male	368 (68.7)	324 (69.2)	44 (64.7)	0.452	1 (Ref)
Female	168 (31.3)	144 (30.8)	24 (35.3)		1.227 (0.719–2.095)
Origin					
Endemic	329 (61.4)	292 (62.4)	37 (54.4)	0.207	1 (Ref)
Non-endemic country	207 (38.6)	176 (37.6)	31 (45.6)		1.390 (0.833–2.321)
Previous malaria episodes					
History of ≥ 1 previous malaria episodes	163 (30.4)	153 (32.7)	10 (14.7)	0.003	1 (Ref)
No history of previous malaria	373 (69.6)	315 (67.3)	58 (85.3)		2.817 (1.401–5.665)
Use of chemoprophylaxis					
Regular use	15 (2.8)	14 (3.0)	1 (1.5)	0.437	1 (Ref)
Irregular/lack of use	426 (79.5)	368 (78.6)	58 (85.3)		2.207 (0.285–17.11)
Missing	95 (17.7)	86 (18.4)	9 (13.2)		
Season					
Fall	116 (21.6)	103 (22.0)	13 (19.1)	0.450	1 (Ref)
Winter	117 (21.8)	100 (21.4)	17 (25.0)		1.35 (0.66–2.92)
WHO region					
African region	508 (94.8)	446 (95.3)	62 (91.2)	0.153	1 (Ref)
Southeast Asian region	16 (3.0)	10 (2.1)	6 (8.8%)	0.003	4.37 (1.53–12.43)
Region of the Americas	4 (0.7)	4 (0.9)	0 (0.0)	–	–
European region	1 (0.2)	1 (0.2)	0 (0.0)	–	–
Eastern Mediterranean region	4 (0.7)	4 (0.8)	0 (0.0)	–	–
Missing	2 (0.4)	2 (0.4)	0 (0.0)	–	–
Reason of travel					
Occupational	92 (17.2)	84 (18.0)	8 (11.8)	0.206	1 (Ref)
Visiting friends and relatives	131 (24.4)	114 (24.4)	17 (25.0)	0.91	1.566 (0.645–3.800)
Tourism	86 (16.0)	66 (14.1)	20 (29.4)	0.001	3.182 (1.318–7.681)
Refugee	2 (0.4)	2 (0.4)	–	–	
Missing	225 (42.0)	202 (43.2)	23 (33.8)		
Patient delay, d					
Median (range)	4 (0–68)	4 (0–68)	5 (0–11)	0.816	1.00 (0.96–1.06)
0–1	26 (4.7)	24 (5.0)	2 (2.4)	0.2919	1 (Ref)
2–3	194 (34.8)	169 (35.6)	25 (30.1)	0.3353	1.78 (0.40–7.98)
≥ 4	255 (45.7)	206 (43.4)	49 (59.0)	0.0082	2.85 (0.65–12.49)
Missing	83 (14.9)	76 (16.0)	7 (8.4)	–	–
Pregnancy					
No (%) of women	11 (6.6)	10 (6.9)	1 (4.2)	0.631	0.60 (0.073–4.905)

*CI* confidence interval, *OR* odds ratio

^a^Estimated by Chi square test for categorical and by Mann–Whitney-U-test for continuous data

^b^Odds ratios (OR) determined by univariate logistic regression

Univariate analysis of the basic sociodemographic parameters revealed that increasing age, lack of previous malaria episodes, presentation delayed for ≥ 4 days, contraction of the disease in the WHO Southeast-Asian region, and being a tourist were associated with severe malaria (Table 2). Information on delay of presentation and reason of travel, however, was missing in a substantial proportion of cases and the association between contraction of the disease in the WHO Southeast-Asian region and severe malaria relied on only 16 subjects. These parameters were therefore not included in the subsequent multivariable analyses.

A total of 95 patients (17.7%) had at least one relevant chronic condition (range 0–3)—the most common being hypertension, endocrine/metabolic disorders, and chronic infectious disorders, including 15 HIV-positive individuals (Table 3). The absolute numbers of co-morbidities as well as a higher seriousness of the underlying disorders (CA-CCI score ≥ 2) were strongly associated with severe malaria in univariate analysis. Hypertension, cardiovascular diseases, dyslipidaemia, malignancy, alcoholism and chronic infections (HIV-infection) were individual diseases associated with severe malaria in univariate analysis. After adjustment for age, endemic origin, and previous malaria episodes as potential confounders the associations remained significant for cardiovascular diseases, hypertension, dyslipidaemia and alcoholism in multivariable logistic regression. For alcoholism, however, model diagnostics revealed presence of significant outliers. Additional adjustment for cardiovascular diseases, dyslipidaemia, and obesity did not affect the association between hypertension and severe malaria (adjusted odds ratios, aOR, 5.90, [95% confidence interval, CI 1.825–19.05].

**Table 3:** Chronic conditions associated with severe imported falciparum malaria in the study population

Chronic co-morbidity	No. (%) of patients			P value^a^	OR (95% CI)	
	Total (n = 536)	Non severe malaria (n = 468)	Severe malaria (n = 68)		Unadjusted^b^	Adjusted^c^
No. of co-morbidities						
0	441 (82.3)	394 (84.2)	47 (69.1)	< 0.002	1 (Ref)	
1	61 (11.4)	50 (10.7)	11 (16.2)	0.183	1.844 (0.898–3.787)	
2	29 (5.4)	20 (4.3)	9 (13.2)	0.002	3.772 (1.624–8.765)	
3	5 (0.9)	4 (0.9)	1 (1.5)	0.625	2.096 (0.229–19.15)	
Seriousness of underlying disorders (CA-CCI)						
≥ 2	110 (20.5)	85 (18.2)	25 (36.8)	< 0.001	2.83 (1.644–4.871)	
Nutritional status						
BMI, median (range)	24.5 (18.0–39.2)	24.6 (18.0–39.2)	24.4 (18.0–34.9)	0.388		
Obesity^d^	24 (9.8)	19 (9.9)	5 (9.4)	0.920	1.054 (0.374–2.970)	
Individual diseases						
Cardiovascular disease	12 (2.2)	5 (1.1)	7 (10.3)	< 0.001	8.961 (2.656–30.234)	8.197 (2.300–29.216)
Hypertension	43 (9.2)	30 (6.4)	13 (19.1)	< 0.001	4.447 (2.233 -8.857)	3.061 (1.335–7.021)
Pulmonary disease	13 (2.4)	12 (2.6)	1 (1.5)	0.567	1.763 (0.226–13.779)	
History of or active smoking	31 (5.8)	24 (5.1)	7 (10.3)	0.088	2.123 (0.878–5.136)	
Endocrine/metabolic disorder^e^	31 (5.8)	26 (5.6)	5 (7.4)	0.553	1.715 (0.677–4.345)	
Diabetes mellitus	16 (3.0)	14 (3.0)	2 (2.9)	0.982	1.615 (0.448–5.822)	
Dyslipidaemia	6 (1.1)	3 (0.6)	3 (4.4)	0.006	7.154 (1.414–36.193)	6.082 (1.125–32.876)
Metabolic syndrome^f^	5 (0.9)	3 (0.6)	2 (2.9)	0.065	4.697 (0.770–28.634)	
Chronic infectious disease^g^	31 (5.8)	23 (4.9)	8 (11.8)	0.024	2.123 (0.878–5.136)	
HIV infection	15 (2.8)	10 (2.1)	5 (7.4)	0.015	2.597 (0.803–8.399)	
Chronic hepatitis	12 (2.2)	9 (1.9)	3 (4.4)	0.195	1.94 (0.51–7.33)	
Malignancy	9 (1.7)	6 (1.3)	3 (4.4)	0.042	3.554 (0.868–14.557)	
Chronic renal disease	10 (1.9)	9 (1.9)	1 (1.5)	0.797	1.314 (0.164–10.535)	
Alcoholism	4 (0.7)	1 (0.2)	3 (4.4)	< 0.001	21.554 (2.209–210.303)	19.217 (1.848–199.870)
Gastrointestinal disorder	4 (0.7)	3 (0.6)	1 (1.5)	0.458	2.313 (0.237–22.564)	
Autoimmune/rheumatoloic disorder	4 (0.7)	3 (0.6)	1 (1.5)	0.458	2.313 (0.237–22.564)	
Neurologic disorder	2 (0.4)	2 (0.4)	–	–	–	

*CA-CCI* age-adjusted Charleson co-morbidity index, *CI* confidence interval, *HIV* human immunodeficiency virus, *OR* odds ratio

^a^Estimated by Chi square test

^b^Odds ratios (OR) determined by univariate logistic regression

^c^Adjusted odds ratios (OR) were determined in separate multivariable models including the individual diagnoses together with age (continuous), origin from endemic region, and lack of previous malaria episodes as potential confounders

^d^Obesity: BMI ≥ 30 kg/m^2^. A BMI was available for 245 cases, 192 with non-severe and 53 with severe malaria

^e^Endocrine/metabolic disorded included diabetes, dyslipidaemia, hypo- and hyperthyroidism, and hyperuricaemia/gout

^f^Metabolic syndrome: obesity together with at least one of the following: hypertension, diabetes, or dyslipidaemia

^g^Chronic infectious disease included infections with HIV, hepatitis B and C, Strongyloides stercoralis, and filarial parasites

**Fig. 1:**
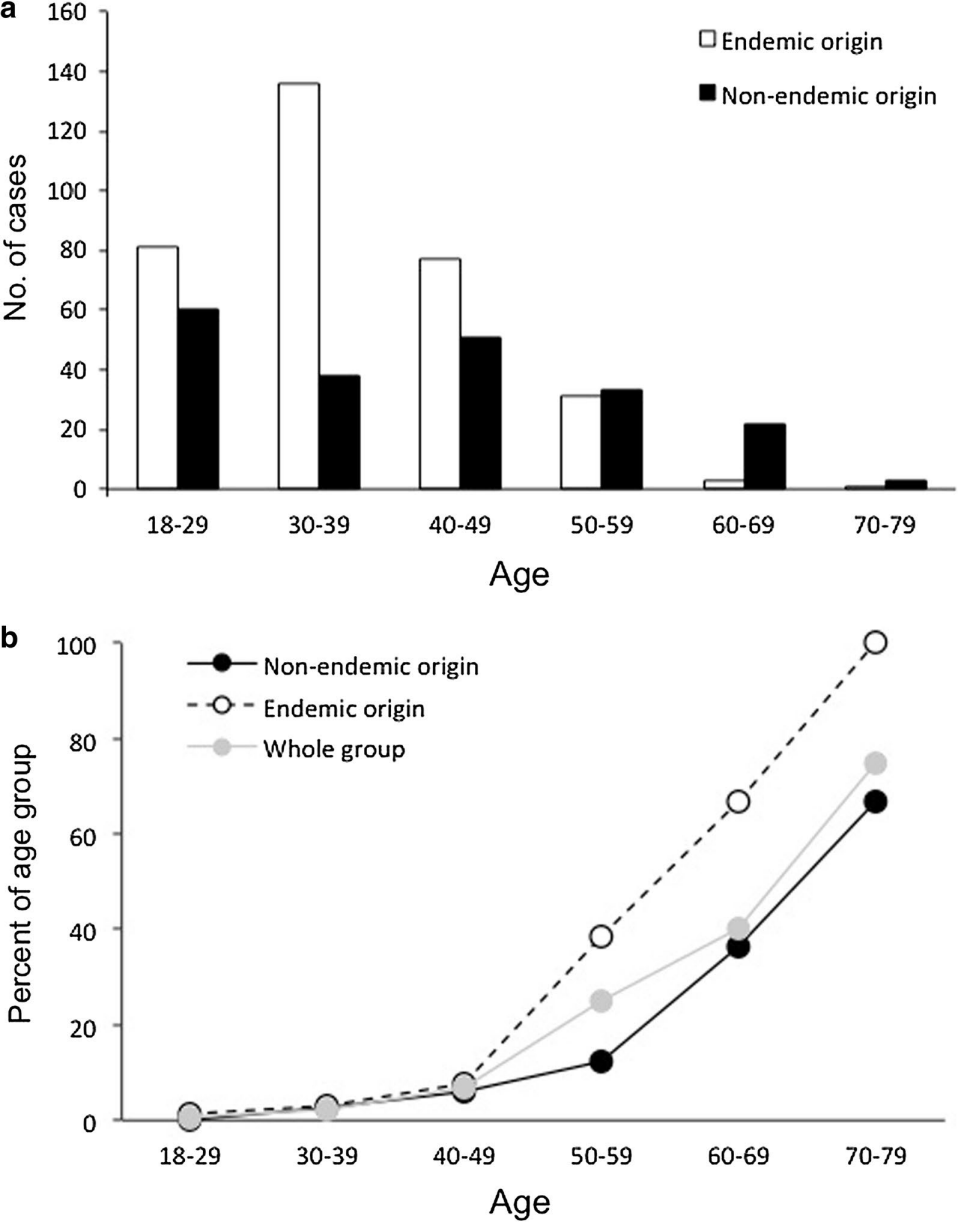
Age distribution (**a**) and prevalence of hypertension (**b**) differed significantly between patients originating from endemic and non-endemic countries. The latter group was older and age distribution was broader. Prevalence of hypertension was strictly age-associated and increased markedly from the fifth decade onwards in both subgroups (**b**). However, hypertension was continuously more prevalent among individuals of endemic origin. Note that only 4 individuals originating from endemic regions and being ≥ 60 years were included

Age distribution differed significantly (p = 0.002) between patients of non-endemic and endemic origin (Fig. 1): among patients of endemic origin median age was 36 years (range 18–78 years) with only 35 individuals (10.6%) being ≥ 50 years of age. The age distribution was broader among patients of non-endemic origin (median age: 41 years; range 18–76 years). In this group, 58 patients (28.0%) were ≥ 50 years of age. Prevalence of hypertension was strictly age-associated and increased markedly from the fifth decade onwards (Fig. 1) in both subgroups. However, hypertension was continuously more prevalent among individuals of endemic origin. After adjustment for age and previous malaria episodes, hypertension was still associated with severe malaria in both subgroups, patients of endemic origin (aOR, 3.60, [95% CI 1.05–12.35]) and non-endemic origin (aOR 4.83, [95% CI 1.44–16.22]), respectively.

## Discussion

The present study demonstrates that hypertension and its secondary diseases increase the risk for a severe course of imported falciparum malaria. The complex pathophysiology of falciparum malaria interferes with the mechanisms that compensate for the systemic effects of the infection on multiple levels. Pre-existing chronic medical conditions can further hamper these physiologic mechanisms, thereby facilitating development of severe malaria. The age-dependent increase in the prevalence of such non-communicable diseases presumably explains to a large extent the association of increasing age with severe falciparum malaria that has consistently been found in various studies.

The prevalence of hypertension is higher in populations constantly exposed to malaria [17]. Therefore, it has been suspected that malaria might be a cause for hypertension in areas of stable transmission and that hypertension confers some degree of protection against malaria as a result of a co-evolutionary process [18]. In Indian adults, polymorphisms in the angiotensin-converting enzyme (ACE) gene, leading to elevated levels of angiotensin II (Ang II) and thus hypertension, have been associated with protection against cerebral malaria [19]. Angiotensin II has subsequently not only been shown to directly inhibit Plasmodium growth in vitro [20]. It also appears to have a protective effect on the blood–brain barrier integrity, presumably by binding to Ang II type 2 (AT2) receptors [21].

In areas of unstable transmission and in imported falciparum malaria, however, the situation is likely different. Numerous studies have demonstrated the diverse effects of falciparum malaria on the cardiovascular system in the setting of acute disease in recent years [22]. These effects have three main components: a reduction of pre-load, direct myocardial suppression and increased after-load.

A reduced pre-load is the result of fluid losses from fever, perspiration, vomiting, diarrhoea, insufficient oral fluid intake, and increased vascular permeability. Nearly all patients with severe falciparum malaria are hypovolemic on presentation [23, 24]. By applying echocardiographic techniques more severe tachycardia, lower stroke volume index, and higher vena cava collapsibility index were found in acidotic malaria patients compared to less severely affected individuals [24]. Increase in heart rate and vasoconstriction are the principal physiologic responses to hypovolaemia.

Direct myocardial impairment has been demonstrated in severe but not in uncomplicated falciparum malaria cases by elevated levels of N-terminal brain natriuretic peptide (NT-pro-BNP) and heart-type fatty acid-binding protein (H-FABP) [25]. Pro-inflammatory cytokines such as tumour necrosis factor (TNF), which are excessively liberated in the context of severe malaria, can directly depress myocardial function [26].

Reduced red-blood cell (RBC) deformability is a pathophysiologic hallmark of falciparum malaria resulting in increased clearance of parasitized and non-parasitized erythrocytes. Cell-free haemoglobin released from ruptured erythrocytes acts as a nitric oxide (NO) scavenger [27], thereby increasing the plasma concentration of asymmetric dimethylarginine (ADMA), an NO synthase inhibitor [28]. Reduced NO bioavailability not only leads to increased pulmonary pressures and myocardial wall stress [29]. It also enhances endothelial dysfunction with subsequently impaired vasodilation [27]. Using invasive haemodynamic monitoring Hanson and colleagues demonstrated higher baseline systemic vascular resistance indexes (SVRI) in patients who died from severe malaria compared to survivors [23]. An increased SVRI together with reduced cardiac output in malaria patients compared to healthy controls could also be demonstrated in a study applying non-invasive techniques [30]. These results correlated with the cardial biomarkers NT-pro-BNP and myoglobin.

**Fig. 2:**
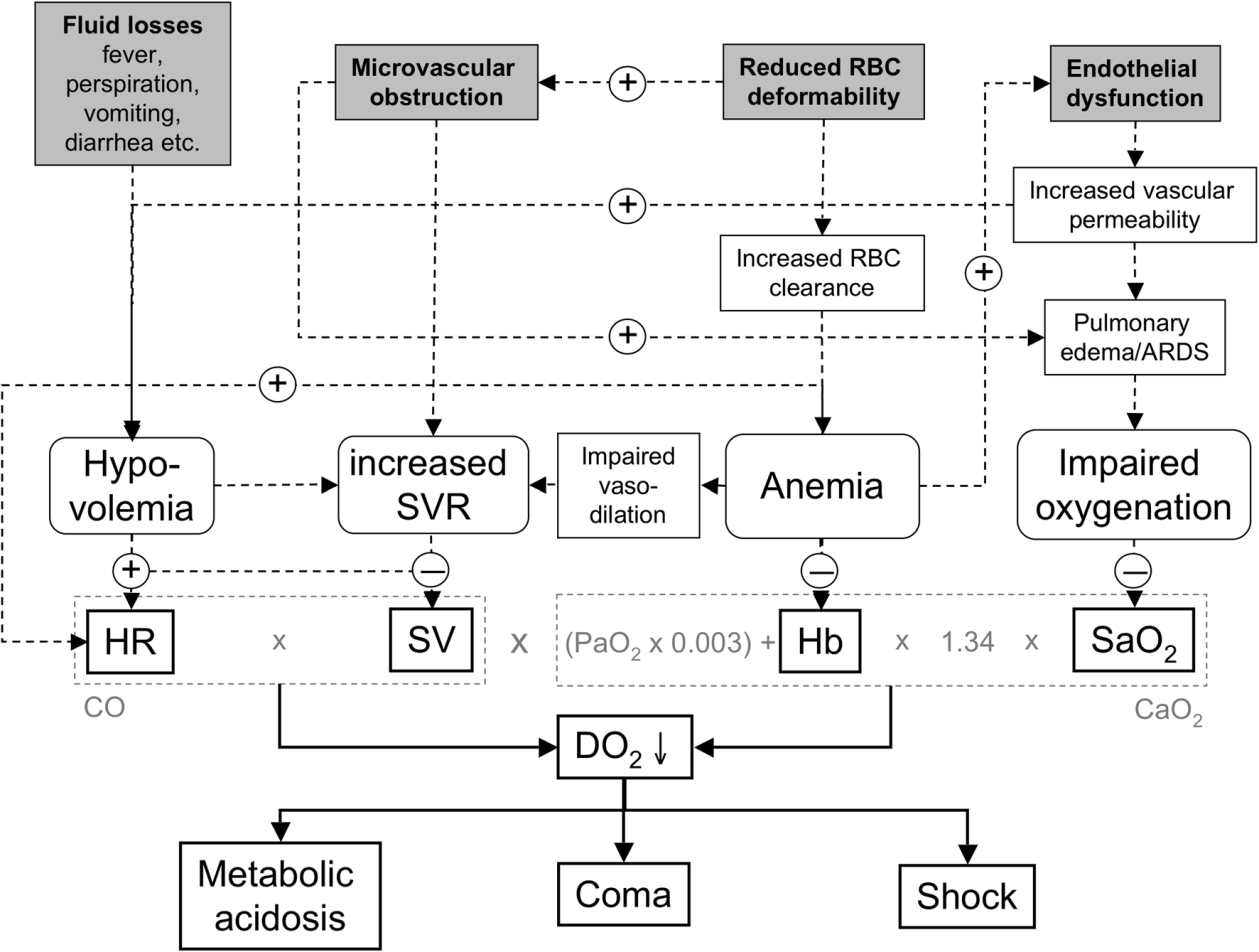
Falciparum malaria pathophysiology interferes with oxygen delivery to peripheral tissues on multiple levels. Oxygen delivery depends on heart rate, stroke volume, haemoglobin level and arterial oxygen saturation. Changes in one parameter can normally be compensated for by others. However, the key elements of falciparum malaria pathophysiology (grey boxes) do not just interfere with all components of oxygen delivery simultaneously, but they may also augment each other. If the capabilities for compensation are limited by pre-existing chronic co-morbidities, development of life-threatening complications such as metabolic acidosis and shock is facilitated. In a recent study from India, for instance, Mohanty et al. demonstrated that clinical and radiologic findings in cerebral malaria are largely consistent with posterior reversible encephalopathy syndrome (PRES) [40]. Hypertension is one of the known etiologies of PRES. Abbreviations: ARDS, acute respiratory distress syndrome; CaO_2_, arterial oxygen content; CO, cardiac output; DO_2_, oxygen delivery; haemoglobin level; HR, heart rate; PaO_2_, partial arterial oxygen pressure; RBC, red blood cell; SaO_2_, arterial oxygen saturation; SV, stroke volume; SVR, systemic vascular resistance

Importantly, the various pathophysiologic processes occur simultaneously and may augment each other, together leading to a reduced oxygen delivery to peripheral tissues and hence complicated malaria (Fig. 2). On the basis of these observations it appears reasonable that pre-existing disorders can add to malaria pathophysiology.

For long, HIV infection is known to increase the risk for severe falciparum malaria [31]. Recently, with diabetes and obesity two non-communicable conditions were identified as risk factors for severe imported falciparum malaria in a nation-wide analysis from Sweden [11]. Among other reasons, the authors explained this by a generally increased severity of infections in obese and diabetic patients [32].

Yet, there are alternative explanations for this association. In malaria pathophysiology obstruction of the microcirculation by parasitized red blood cells plays a central role [33]. Pre-existing microangiopathy could thus add to the deleterious effects of microcirculatory blood flow obstruction in acute falciparum malaria. Diabetes mellitus, lipid disorders, and hypertension are key factors for the development of microangiopathy. Arterial hypertension induces two types of diffuse structural changes in the systemic microcirculation: rarefaction and remodelling. Rarefaction refers to an abnormally reduced density of arterioles, capillaries and possibly venules [34]. Remodelling leads to a structural reduction in lumen diameter of resistance small arteries and arterioles [35]. While both structural modifications are seen in hypertensive patients, remodelling seems to play a paramount role in long-term elevation of systemic vascular resistance. In addition to these structural changes, hypertension as well as diabetes induces functional changes in the microvasculature. Hypertension- and diabetes-related endothelial dysfunction leads to reduced bioavailability of local vasodilators (such as NO and prostacyclin) and increased formation of vasoconstrictors and reactive oxygen species, balancing the microvasculature towards vasoconstriction. These functional microvascular changes result in increased systemic vascular resistance and reduced oxygen delivery to target organs [36]. Pre-existing hypertension- and diabetes-associated microangiopathy may thus potentiate the reduced oxygen supply to vital organs caused by imported falciparum malaria, thereby facilitating severe forms of the disease (Additional file 1).

Similar to diabetes and hypertension lipid disorders promote development of microangiopathy [37]. Hypertension-related cardiovascular diseases such as congestive heart failure, ischemic heart disease or atrial fibrillation likely contribute themselves to a decreased cardiac output in the setting of acute infection as depicted in Fig. 2.

In addition to various well-characterized risk factors for development of severe falciparum malaria such as increasing age, lack of semi-immunity (no history of previous malaria episodes, being a tourist), or delayed presentation chronic co-morbidity was associated with complicated disease in the present cohort. Not only the number of chronic conditions but also the seriousness of underlying co-morbidity (assessed by age-adjusted Charleson co-morbidity index) influenced the risk for development of severe falciparum malaria. One of the individual diagnoses responsible for this association was hypertension. With a prevalence of 9.2% hypertension was the single most common chronic condition in the study population. Multivariable analysis was therefore robust and revealed that hypertensive individuals had a threefold increased risk of developing severe imported falciparum malaria compared to patients without hypertension. With increased after-load, hypertensive cardiomyopathy and, most importantly, pre-existing microangiopathy, there were plausible pathophysiologic explanations for this association (Additional file 2). The epidemiologic heterogeneity of the cohort, however, required a more detailed interpretation of the results. As expected, prevalence of hypertension was higher among patients of endemic origin. Simultaneously, median age was significantly lower in this subgroup, with only 4 patients being ≥ 60 years. Yet, hypertension appeared to be independently associated with development of severe malaria regardless of origin.

The much lower case numbers for individuals with cardiovascular diseases, lipid disorders und alcoholism warranted a more careful interpretation of the association of these conditions with severe malaria. Yet—although statistically not significant in the final multivariable model—there was also a strong trend towards an increased risk for severe disease in patients with cardiovascular diseases in the study of Wyss et al. [11]. Besides, older age, alcohol use, diabetes, atrial fibrillation, dyslipidaemia and hypertension have also been associated with an increased risk for sepsis [38], a condition that shares certain features with falciparum malaria. Similar to the findings of the present study, the risk of sepsis increases with the number chronic medical conditions.

These findings have implications for supportive therapy of imported falciparum malaria. Effective anti-malarials usually achieve complete parasite clearance within 96 h of initiation. Aim of supportive therapy is to prevent the patient from harm due to the systemic effects of the disease. This is of particular importance in the prognostically decisive first 48 h of treatment. Yet, endothelial activation and inflammation can persist for up to 28 days after therapy (the so-called “post-treatment inflammatory effect“) [39]. The supportive therapy must, therefore, meet the specific pathophysiologic requirements of falciparum malaria well beyond completion of antimalarial therapy. Support of the cardiovascular system in severe malaria can be provided by numerous interventions such as correction of hypovolaemia by careful rehydration, controlling tachycardia in atrial fibrillation, maintaining adequate haemoglobin levels in order to ensure sufficient oxygenation—especially in concomitant coronary artery disease—or by providing inotropes in myocardial failure. The data presented here indicate that reducing an increased after-load with appropriate antihypertensives is another important intervention. As outlined elsewhere [18], Angiotensin-I antagonists are probably superior to other substances in this context. In circulatory shock and acute kidney injury, however, their use should be avoided.

The present study has several limitations, the main being its retrospective nature. Due to misclassification of exposure, for example, the medical histories of patients with severe disease could have been more thoroughly investigated than those of uncomplicated cases. However, all patients enrolled were hospitalized, treated under identical conditions, and their courses were recorded in standardized electronic files. Thus, data capture was high. Long observation period and monocentric design further add to limited quality of the data. However, severe imported falciparum malaria is a rare condition in most non-endemic regions. Thus, high-quality prospective trials conducted in resource-rich settings will hardly become available in the future. Retrospective analyses, therefore, continue to provide an important source of evidence. Together with a case load of > 500 individuals this allowed for detailed statistical analyses. Since cohorts of patients with imported falciparum malaria will differ in regard to average age, represented ethnicities, immune status, comorbidities, destinations, reason of travel and other important aspects, however, the results cannot be generalized recklessly. Though obesity and diabetes did not prove risk factors in the present cohort the analysis nevertheless strongly supports the hypothesis that chronic medical conditions promoting microangiopathy or reduced cardiac output facilitate development of severe falciparum malaria.

## Conclusions

In addition to diabetes and obesity, hypertension is another previously unidentified risk factor for severe disease in adults with imported falciparum malaria. Due to its high prevalence this finding is not only of importance for travellers of non-endemic origin but also for expatriates originating from endemic areas with slowly waning semi-immunity. The study also gave evidence that related conditions such as cardiovascular diseases or lipid disorders may increase the risk for development of severe disease. Based on pathophysiologic considerations these associations are plausible.

## Supplementary information

**Additional file 1.** Severity criteria among hypertensive and non-hypertensive patients with severe falciparum malaria imported to Berlin, Germany.

**Additional file 2.** Prevalence of selected co-morbidities among 536 patients with and without hypertension and falciparum malaria imported to Berlin, Germany.

## Data Availability

The datasets used and analysed during the current study are available from the corresponding author on reasonable request.

## Abbreviations

ACE: angiotensin-converting enzyme
ADMA: asymmetric dimethylarginine
Ang II: angiotensin II
ARDS: acute respiratory distress syndrome
AT2 receptors: angiotensin-II type 2 receptors
CA-CCI: age-adjusted Charleson co-morbidity index
CaO_2_: arterial oxygen content
CO: cardiac output
DO_2_: oxygen delivery
GCS: Glasgow coma scale
GPI: glycosylphosphatidylinositol
Hb: haemoglobin level
H-FABP: Heart-type fatty-acid binding protein
HIV: Human immunodeficiency virus
HR: Heart rate
NO: nitirc oxide
NT-pro-BNP: N-terminal brain natriuretic peptide
PaO_2_: partial arterial oxygen pressure
RBC: red-blood cell
SaO_2_: arterial oxygen saturation
SV: stroke volume
SVRI: systemic vascular resistance index
TNF: tumour necrosis factor
VFR: visiting friends and relatives
WHO: World Health Organization

## References

[1] Schlagenhauf P, Weld L, Goorhuis A, Gautret P, Weber R, von Sonnenburg F (2014). Travel-associated infection presenting in Europe (2008–12): an analysis of EuroTravNet longitudinal, surveillance data, and evaluation of the effect of the pre-travel consultation. Lancet Infect Dis..

[2] Miller LH, Ackerman HC, Su XZ, Wellems TE (2013). Malaria biology and disease pathogenesis: insights for new treatments. Nat Med.

[3] Bernabeu M, Smith JD (2016). EPCR and malaria severity: the center of a perfect storm. Trends Parasitol..

[4] Simpson JA, Aarons L, Collins WE, Jeffery GM, White NJ (2002). Population dynamics of untreated *Plasmodium falciparum* malaria within the adult human host during the expansion phase of the infection. Parasitology.

[5] WHO (2014). Guidelines for the treatment of malaria.

[6] Luthi B, Schlagenhauf P (2014). Risk factors associated with malaria deaths in travellers: a literature review. Travel Med Infect Dis..

[7] Schwartz E, Sadetzki S, Murad H, Raveh D (2001). Age as a risk factor for severe *Plasmodium falciparum* malaria in nonimmune patients. Clin Infect Dis.

[8] Legros F, Bouchaud O, Ancelle T, Arnaud A, Cojean S, Le Bras J (2007). Risk factors for imported fatal *Plasmodium falciparum* malaria, France, 1996–2003. Emerg Infect Dis.

[9] Krause G, Schoneberg I, Altmann D, Stark K (2006). Chemoprophylaxis and malaria death rates. Emerg Infect Dis.

[10] Checkley AM, Smith A, Smith V, Blaze M, Bradley D, Chiodini PL (2012). Risk factors for mortality from imported falciparum malaria in the United Kingdom over 20 years: an observational study. BMJ.

[11] Wyss K, Wangdahl A, Vesterlund M, Hammar U, Dashti S, Naucler P (2017). Obesity and diabetes as risk factors for severe *Plasmodium falciparum* malaria: results from a Swedish nationwide study. Clin Infect Dis.

[12] Allen N, Bergin C, Kennelly SP (2016). Malaria in the returning older traveler. Trop Dis Travel Med Vaccines..

[13] Lim SS, Vos T, Flaxman AD, Danaei G, Shibuya K, Adair-Rohani H (2013). A comparative risk assessment of burden of disease and injury attributable to 67 risk factors and risk factor clusters in 21 regions, 1990–2010: a systematic analysis for the Global Burden of Disease Study 2010. Lancet.

[14] Bruneel F, Tubach F, Corne P, Megarbane B, Mira JP, Peytel E (2010). Severe imported falciparum malaria: a cohort study in 400 critically ill adults. PLoS ONE.

[15] Sundararajan V, Henderson T, Perry C, Muggivan A, Quan H, Ghali WA (2004). New ICD-10 version of the Charlson comorbidity index predicted in-hospital mortality. J Clin Epidemiol.

[16] Stoltzfus JC (2011). Logistic regression: a brief primer. Acad Emerg Med.

[17] Danaei G, Finucane MM, Lin JK, Singh GM, Paciorek CJ, Cowan MJ (2011). National, regional, and global trends in systolic blood pressure since 1980: systematic analysis of health examination surveys and epidemiological studies with 786 country-years and 5.4 million participants. Lancet..

[18] Gallego-Delgado J, Walther T, Rodriguez A (2016). The high blood pressure-malaria protection hypothesis. Circ Res.

[19] Dhangadamajhi G, Mohapatra BN, Kar SK, Ranjit M (2010). Gene polymorphisms in angiotensin I converting enzyme (ACE I/D) and angiotensin II converting enzyme (ACE2 C– > T) protect against cerebral malaria in Indian adults. Infect Genet Evol..

[20] Saraiva VB, de Souza Silva L, Ferreira-DaSilva CT, da Silva-Filho JL, Teixeira-Ferreira A, Perales J (2011). Impairment of the *Plasmodium falciparum* erythrocytic cycle induced by angiotensin peptides. PLoS ONE.

[21] Gallego-Delgado J, Basu-Roy U, Ty M, Alique M, Fernandez-Arias C, Movila A (2016). Angiotensin receptors and beta-catenin regulate brain endothelial integrity in malaria. J Clin Invest..

[22] Mishra SK, Behera PK, Satpathi S (2013). Cardiac involvement in malaria: an overlooked important complication. J Vector Borne Dis..

[23] Hanson JP, Lam SW, Mohanty S, Alam S, Pattnaik R, Mahanta KC (2013). Fluid resuscitation of adults with severe falciparum malaria: effects on acid-base status, renal function, and extravascular lung water. Crit Care Med.

[24] Yacoub S, Lang HJ, Shebbe M, Timbwa M, Ohuma E, Tulloh R (2010). Cardiac function and hemodynamics in Kenyan children with severe malaria. Crit Care Med.

[25] Ehrhardt S, Wichmann D, Hemmer CJ, Burchard GD, Brattig NW (2004). Circulating concentrations of cardiac proteins in complicated and uncomplicated *Plasmodium falciparum* malaria. Trop Med Int Health..

[26] Prabhu SD (2004). Cytokine-induced modulation of cardiac function. Circ Res.

[27] Yeo TW, Lampah DA, Tjitra E, Gitawati R, Kenangalem E, Piera K (2009). Relationship of cell-free hemoglobin to impaired endothelial nitric oxide bioavailability and perfusion in severe falciparum malaria. J Infect Dis.

[28] Yeo TW, Lampah DA, Tjitra E, Gitawati R, Darcy CJ, Jones C (2010). Increased asymmetric dimethylarginine in severe falciparum malaria: association with impaired nitric oxide bioavailability and fatal outcome. PLoS Pathog.

[29] Janka JJ, Koita OA, Traore B, Traore JM, Mzayek F, Sachdev V (2010). Increased pulmonary pressures and myocardial wall stress in children with severe malaria. J Infect Dis.

[30] Herr J, Mehrfar P, Schmiedel S, Wichmann D, Brattig NW, Burchard GD (2011). Reduced cardiac output in imported *Plasmodium falciparum* malaria. Malar J..

[31] Cohen C, Karstaedt A, Frean J, Thomas J, Govender N, Prentice E (2005). Increased prevalence of severe malaria in HIV-infected adults in South Africa. Clin Infect Dis.

[32] Falagas ME, Kompoti M (2006). Obesity and infection. Lancet Infect Dis..

[33] Dondorp AM, Kager PA, Vreeken J, White NJ (2000). Abnormal blood flow and red blood cell deformability in severe malaria. Parasitol Today..

[34] Feihl F, Liaudet L, Waeber B, Levy BI (2006). Hypertension: a disease of the microcirculation?. Hypertension.

[35] Park JB, Schiffrin EL (2001). Small artery remodeling is the most prevalent (earliest?) form of target organ damage in mild essential hypertension. J Hypertens.

[36] Nyberg M, Gliemann L, Hellsten Y (2015). Vascular function in health, hypertension, and diabetes: effect of physical activity on skeletal muscle microcirculation. Scand J Med Sci Sports.

[37] Stapleton PA, Goodwill AG, James ME, Brock RW, Frisbee JC (2010). Hypercholesterolemia and microvascular dysfunction: interventional strategies. J Inflamm (Lond).

[38] Wang HE, Shapiro NI, Griffin R, Safford MM, Judd S, Howard G (2012). Chronic medical conditions and risk of sepsis. PLoS ONE.

[39] Moxon CA, Chisala NV, Wassmer SC, Taylor TE, Seydel KB, Molyneux ME (2013). Persistent endothelial activation and inflammation after *Plasmodium falciparum* infection in Malawian children. J Infect Dis.

[40] Mohanty S, Benjamin LA, Majhi M, Panda P, Kampondeni S, Sahu PK (2017). Magnetic resonance imaging of cerebral malaria patients reveals distinct pathogenetic processes in different parts of the brain. mSphere..

