# Supplementary material for: Hypertension is associated with an increased risk for severe imported falciparum malaria: a tertiary care hospital based observational study from Berlin, Germany
Source: Malar J. 2019 Dec 6;18:410. doi: 10.1186/s12936-019-3007-4 (PMC6898961; doi:10.1186/s12936-019-3007-4)
Supplement: Supplementary file 1 — Additional file 1. Severity criteria among hypertensive and non-hypertensive patients with severe falciparum malaria imported to Berlin, Germany. [file 12936_2019_3007_MOESM1_ESM.doc]

**Additional file 1** Severity criteria among hypertensive and non-hypertensive patients with severe falciparum malaria imported to Berlin, Germany

| Severity criterion | Hypertensive  (n=13) | Non-hypertensive  (n= 55) | P Value |
| --- | --- | --- | --- |
| GCS<11, No (%) | 2 (15.4) | 4 (7.3) | 0.25 |
| >2 convulsions, No (%) | 0 (0) | 0 (0) | - |
| Respiratory distress, No (%) | 1 (7.7) | 4 (7.3) | 0.96 |
| Circulatory collapse, No (%) | 4 (30.8) | 5 (9.1) | 0.04 |
| APO, No (%) | 2 (15.4) | 5 (9.1) | 0.50 |
| ARDS, No (%) | 1 (7.7) | 1 (1.8) | 0.26 |
| AKIN, No (%) | 2 (15.4) | 3 (5.5) | 0.22 |
| Acidosis, No (%) | 3 (23.1) | 6 (10.9) | 0.24 |
| Jaundice, No (%) | 2 (15.4) | 8 (14.6) | 0.94 |
| Anaemia, No (%) | 1 (7.7) | 4 (7.3) | 0.96 |
| Haemoglobinuria, No (%) | 1 (7.7) | 10 (18.2) | 0.36 |
| Bleeding, No (%) | 2 (15.4) | 7 (12.7) | 0.80 |
| Hypoglycemia, No (%) | 0 (0) | 1 (1.8) | 0.62 |
| Hyperparasitemia, No (%) | 10 (77.0) | 52 (94.6) | 0.044 |

Abbreviations: APO, acute pulmonary oedema; AKIN, acute kidney injury; ARDS, acute respiratory distress syndrome; GCS, Glasgow coma scale.
