# Supplementary material for: Hypertension is associated with an increased risk for severe imported falciparum malaria: a tertiary care hospital based observational study from Berlin, Germany
Source: Malar J. 2019 Dec 6;18:410. doi: 10.1186/s12936-019-3007-4 (PMC6898961; doi:10.1186/s12936-019-3007-4)
Supplement: Supplementary file 2 — Additional file 2. Prevalence of selected co-morbidities among 536 patients with and without hypertension and falciparum malaria imported to Berlin, Germany. [file 12936_2019_3007_MOESM2_ESM.doc]

**Additional file 2 Prevalence of selected co-morbidities among 536 patients with and without hypertension and falciparum malaria imported to Berlin, Germany**

| Co-morbidity | Hypertension  (n=43) | No hypertension  (n=493) | P Value |
| --- | --- | --- | --- |
| Cardiovascular diseases^1^, No (%) | 9 (20.1) | 3 (0.6) | <0.0001 |
| Dyslipidaemia, No (%) | 2 (4.7) | 4 (0.8) | P=0.022 |
| Alcoholism, No (%) | 0 (0) | 4 (0.8) | - |
| Diabetes, No (%) | 9 (20.1) | 7 (1.4) | <0.0001 |
| Obesity^2^, No (%) | 4 (9.3) | 20 (4.1) | 0.111 |

^1^Includes atrial fibrillation (n=3), congestive heart failure (n=2), coronary artery disease (n=3), peripheral vascular disease (n=2), cerebrovascular disease (n=1), and thrombembolism (n=1).

^2^Data on BMI were available for 245 patients.

Abbreviations: BMI, body mass index.
